# Human Health Risk Assessment of Heavy Metal Concentration in Seafood Collected from Pattani Bay, Thailand

**DOI:** 10.3390/toxics11010018

**Published:** 2022-12-26

**Authors:** Phanwimol Tanhan, Niyada Lansubsakul, Napasorn Phaochoosak, Pattanasuda Sirinupong, Pun Yeesin, Kanjana Imsilp

**Affiliations:** 1 Department of Pharmacology, Faculty of Veterinary Medicine, Kasetsart University, Bangkok 10900, Thailand; 2 Department of Anatomy, Faculty of Veterinary Medicine, Kasetsart University, Bangkok 10900, Thailand; 3 Department of Science, Faculty of Science and Technology, Prince of Songkla University, Pattani Campus, Pattani 94000, Thailand; 4 Department of Agricultural and Fishery Science, Faculty of Science and Technology, Prince of Songkla University, Pattani Campus, Pattani 94000, Thailand

**Keywords:** emerging pollutants, health risk assessment, heavy metals, Pattani Bay, seafood

## Abstract

A significant impact of marine pollution is the contamination of seafood which has raised concerns due to its potential human health risks. This current study investigated seasonal bioaccumulation of 9 heavy metals (Cd, Co, Cr, Cu, Fe, Mn, Ni, Pb, and Zn) in 14 commercially important seafood species, including 4 fish, 5 molluscs, and 5 crustacean species. Samples were collected from Pattani Bay, Pattani province, Thailand, during the dry (July 2020) and wet (February 2021) seasons. The edible samples were analyzed for heavy metal concentrations using a flame atomic absorption spectrophotometer. The bioaccumulation trend of heavy metals decreased in the sequence of molluscs > crustaceans > fish. The possible human health risks associated with heavy metal-contaminated seafood consumption were assessed. The parameters investigated for non-carcinogenic and carcinogenic were target hazard quotient (THQ), total hazard index (HI), and target cancer risk (TR). The average ranges of THQs (7.79 × 10^−8^–8.97 × 10^−3^), HIs (4.30 × 10^−5^–1.55 × 10^−2^), and TRs (2.70 × 10^−9^–1.34 × 10^−5^) were observed in the studied seafood species. The results revealed no non-carcinogenic and carcinogenic health risks from consuming these 14 kinds of seafood.

## 1. Introduction

Heavy metals are released into the environment by weather and rock erosion as their natural sources. These metals enter marine and coastal environments through different anthropogenic and lithogenic activities [1,2]. Dredging is one of the primary anthropogenic sources of heavy metal contamination in estuaries and bays [3]. Once they enter the aquatic environment, heavy metals can easily be assimilated and accumulated in organisms which could cause subsequent detrimental effects. Pattani Bay, located in Pattani province, Southern Thailand, is a semi-enclosed bay that opens to the Gulf of Thailand. It is one of the most critical aquacultural bays in Thailand. The International Union for the Conservation of Nature identified Pattani Bay as a worthy coastal wetland for conservation in Asia. The wealthy and good geographic location of Pattani Bay has created many activities, particularly economic activities such as industrial-zone expansion, coastal aquaculture, seafood-bank establishment, and seaport development. These activities need significant volumes of natural resources and can subsequently generate great magnitude of environmental pollution. Excess sediment from aquaculture farming, heavy-metal contamination from mining, and sewage from urban areas, as well as wastewater from industrial estates, can occur [4]. Regular dredging is thus needed for Pattani Bay to maintain the access of large vessels. All mentioned activities could increase pollutant concentrations including heavy metals in the bay. They can eventually enter the food chain. The numbers of heavy metals (As, Cd, Cr, Cu, Mn, Ni, Pb, and Zn) have been reported in sediment and water collected from Pattani Bay [5,6]. However, only a few studies were conducted to investigate both heavy metal contamination and marine species residing in the area.

Seafood is an essential source of nutrients for coastal residents. It provides proteins, carbohydrates, and vitamins, as well as minerals for human beings [1,7,8]. Worldwide seafood consumption has long been increasing, with a growing interest in nutritional and health benefits [9]. The occurrence of contaminants, which can cause potential human health risks including heavy metals in seafood, have been widely reported [10,11,12,13]. With regard to human health, toxic metals such as Cd, Co, Cr, Ni, and Pb accumulated in seafood can cause neurological disorders, kidney damage, circulatory system problems, and an increased risk of cancer [14]. Other metals such as Cu, Fe, Mn, Ni, and Zn are essential for normal human cellular functions at a specific range of cellular concentrations. A high intake of essential metals can also lead to toxic effects, whereas low intake would cause deficiency disorders [14,15]. Several marine species were used as biological indicators to assess adverse human health effects from consuming contaminated marine organisms. Most species monitored were fish, molluscs, and crabs which are commercially available [10,16,17,18].

This study aimed to investigate 9 heavy metal (Cd, Co, Cr, Cu, Fe, Mn, Ni, Pb, and Zn) concentrations in 14 seafood species (4 fish, 5 molluscs, and 5 crustaceans) collected from Pattani Bay, during the dry (July 2020) and wet (February 2021) seasons. Human health risks were also estimated for non-carcinogenic and carcinogenic threats from consuming these possible heavy metal-contaminated seafood.

## 2. Materials and Methods

### 2.1. Sample Collection

**Figure 1:**
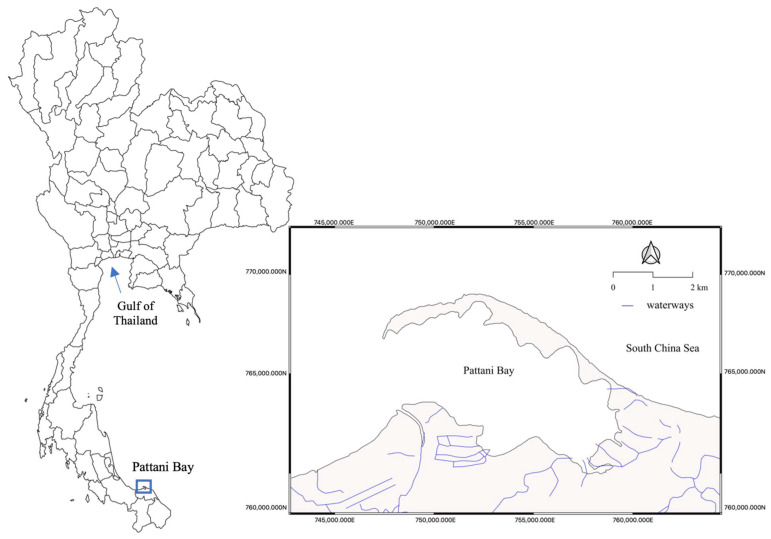
Map of Pattani Bay, Pattani province, Thailand.

Fourteen seafood species were collected in the dry (July 2020) and wet (February 2021) seasons from Pattani Bay, Pattani province, Thailand (Figure 1). Those included four species of fish. Namely, flathead grey mullet, (*Mugil cephalus*, *n* = 45); giant catfish, (*Netuma thalassina*, *n* = 21); striped eel-catfish (*Plotosus lineatus*, *n* = 21); and spotted scat, (*Scatophagus argus*, *n* = 15). In addition, five species of molluscs Pacific oyster, (*Magallana gigas*, *n* = 15); Asiatic hard clam, (*Meretrix meretrix* (L.), *n* = 30); Asian green mussel, (*Perna viridis*, *n* = 60); common geloina, (*Polymesoda erosa*, *n* = 45); and blood cockle, (*Tegillarca granosa*, *n* = 60)]. Five species of crustacean [banana shrimp, (*Fenneropenaeus merguiensis* (de Man, 1888), *n* = 45); giant tiger prawn, (*Penaeus monodon*, *n* = 39); blue swimming crab, (*Portunus pelagicus*, *n* = 15); orange mud crab, (*Scylla olivacea*, *n* = 30); and green mud crab, (*S. paramamosain*, *n* = 30)] [19,20,21,22,23,24]. These seafood species were preferred since they are the favorite species consumed by Thai people and foreign tourists. All samples were chilled, packed, and transported to the laboratory. The edible portions of samples were dissected, homogenized, weighed, and then immediately stored at −20 °C until further analysis.

### 2.2. Heavy Metal Analysis

Samples for heavy metal analysis were oven-dried at 60 °C, and dried sample weights were recorded. Each dried sample (0.5 g) was weighed in triplicate and digested with 5 mL of a mixture of highly purified concentrated nitric acid (69%): hydrogen peroxide (30%) (2:1) at 120 °C until the colour of the solutions was pale yellow and clear. After digestion was complete and adequately cooled down, solutions were filtered through a No. 4 Whatman^®^ filter paper and the volume made up to 25 mL with ultrapure water (18.2 mΩ/cm^2^ Milli-Q Water) for metal analysis. Nine heavy metals (Cd, Co, Cr, Cu, Fe, Mn, Ni, Pb, and Zn) were determined using a flame atomic absorption spectrophotometer (FAAS; SpectrAA 240B Agilent technologies, Victoria, Australia) equipped with a deuterium background corrector. All metals were analyzed in an absorbance mode at the optimal wavelength for each metal: Cd 228.8 mm, Co 240.7 mm, Cr 357.9 nm, Cu 324.7 nm, Fe 248.3 nm, Mn 279.5 nm, Ni 232.0 nm, Pb 217.0 nm, and Zn 213.9 nm. The atomisation was conducted in an air/acetylene flame at 13.3–2.9 L/min for Cr and 13.5–2.0 L/min for other elements. The intensities of the hallow cathode lamp current were 4.0 mA (Cd, Cu, and Ni), 5.0 mA (Fe, Mn, Pb, and Zn), and 7.0 mA (Co and Cr). The individual metal concentration was calculated using its corresponding calibration curve. The permissibility of metal concentrations in foodstuffs, recommended by various organizations including CODEX, EU, FAO/WHO, and Thailand [25,26,27,28,29,30,31], have been reported on a wet weight basis. To make a fair comparison of the determined metal concentrations, all data were converted to their wet weight using the following Equation (1) [32]:

(1) $$C_{\mathrm{ww}}=\frac{(100\;-{\;W}_{s})}{100}{\;\times \;C}_{\mathrm{dw}}$$

where C_ww_ is metal concentration in wet weight (mg/kg ww); W_s_ is water in the sample (%); and C_dw_ is metal concentration in dry weight (mg/kg dw).

The calibration curve linearity (r^2^ > 0.999) was evaluated using seven replicates of six concentrations of each metal. Metal concentrations ranged from 0.05–2.00 μg/g for Cd, 0.05–5.00 μg/g for Co and Cu, 0.10–10.00 μg/g for Cr, 0.05–4.50 μg/g for Fe, 0.25–4.00 μg/g for Mn, 0.50–10.00 μg/g for Ni, 0.50–20.00 μg/g for Pb, and 0.10–2.00 μg/g for Zn. The percentage recovery of each heavy metal was determined based on blank and certified referenced materials for mussel tissue (EMR-CE278k). They were greater than 97.5% for all studied metals. All tests had precision (%RSD) below 10% (0.07–4.68%). The limits of detections (LODs) were the concentrations that the instrument could detect and were calculated as three times the standard deviation of blanks for each heavy metal. The limits of quantification (LOQs) were the concentrations that the instrument could detect and quantify and were calculated as ten times the standard deviation of blanks. The LODs and LOQs of all heavy metals ranged from 0.0005–0.0020 µg/g and 0.001–0.005 µg/g, respectively.

### 2.3. Health Risk Assessment of Heavy Metals

Target hazard quotients (THQs) and target cancer risk (TR) were used to evaluate human health risks from metal-contaminated seafood consumption.

#### 2.3.1. Non-Carcinogenic Risk

The non-carcinogenic health risk of each heavy metal was assessed using the target hazard quotient (THQ). THQ is the ratio of the chronic daily intake (CDI) divided by the oral reference dose (RfD) of individual heavy metals. The RfD assumes that thresholds exist for specific toxic effects. It is an estimate of the daily exposure of the human population that is unlikely to affect human health for a lifetime. The RfD values used to determine the THQ were shown in Table 1 [31,33,34,35,36], whereas the following equation estimated the CDI (2):

(2) $$\mathrm{CDI}=\frac{C\;\times \;\mathrm{EF}\;\times \;\mathrm{ED}\;\times \;\mathrm{IR}}{\mathrm{AT}\;\times \;\mathrm{LT}\;\times \;\mathrm{BW}}$$

where C is the average heavy metal concentration in seafood (mg/kg ww); EF is the exposure frequency (365 days/year); ED is the exposure duration (Thai life expectancy 77.7 years) [37]; IR is the ingestion rate (seafood consumption rate; mg/person/day) [38]; AT is the average time (365*ED; days); LT is the lifetime (equal to exposure duration; years); and BW is an average body weight of Thai men (57 kg) and women (50 kg) [37].

The THQ of each heavy metal was used to assess non-carcinogenic human health risks. It was calculated using the following Equation (3):

(3) $$\mathrm{THQ}=\frac{\mathrm{CDI}}{\mathrm{RfD}}$$

If the THQ value of the heavy metal was less than or equal to 1, it was assumed not to pose non-carcinogenic human health risk over lifetime exposure [38]. On the contrary, if it is more than one, the specific metal can cause non-carcinogenic risk in humans. All calculated THQ values for heavy metals were added to yield a total hazard index (HI). These HIs were used to determine the non-carcinogenic risk of multiple heavy metals. It was calculated using the following Equation (4):

(4) $$\mathrm{HI}\;=\overset{n}{\underset{i=1}{\sum }}{\mathrm{THQ}}_{i}$$

#### 2.3.2. Carcinogenic Risk

The carcinogenic risks were estimated as the cumulative probability of an individual developing cancer over lifetime exposure to that potential carcinogen. Carcinogenic health risks related to the consumption of seafood were measured based on target cancer risk (TR) and were calculated as follows (5):

(5) $${\mathrm{TR}=\mathrm{EDI}\;\times \;\mathrm{CSF}\;\times \;10}^{-3}$$

where CSF is the oral carcinogenic slope factor (mg/kg-day) from the Integrated Risk Information System [36]. The CSF values of selected heavy metals used in carcinogenic risk analysis are shown in Table 1. The EDI is the estimated daily intake of heavy metals from seafood consumption depending on the heavy metal concentrations in seafood and the amount consumed. The EDI was calculated using the following Equation (6):

(6) $$\mathrm{EDI}=\frac{C\;\times \;\mathrm{IR}}{\mathrm{BW}}$$

where C is the average heavy metal concentration in seafood (mg/kg ww); IR is the ingestion rate (seafood consumption rate; mg/person/day) [38]; and BW is an average body weight of Thai men (57 kg) and women (50 kg) [37].

### 2.4. Statistical Analysis

The one-way analysis of variance (one-way ANOVA) and paired sample *t*-test were used to determine differences in the accumulation and distribution of heavy metals in seafood species between seasons (dry and wet seasons). A statistically significant difference was set at *p* < 0.05. Correlations between heavy metals were demonstrated to reflect sediment contamination’s possibly similar chemical properties. The principal component analyses (PCA) are mathematical procedures used to identify a few components by converting a set of correlated variables into uncorrelated variables. The PCA was used to identify the relationship of heavy metals accumulation in seafood tissue and season. All statistical analyses were conducted using SPSS v.23.0 (IBM, Chicago, IL, USA).

## 3. Results and Discussion

### 3.1. Heavy Metal Concentrations in Seafood

**Figure 2:**
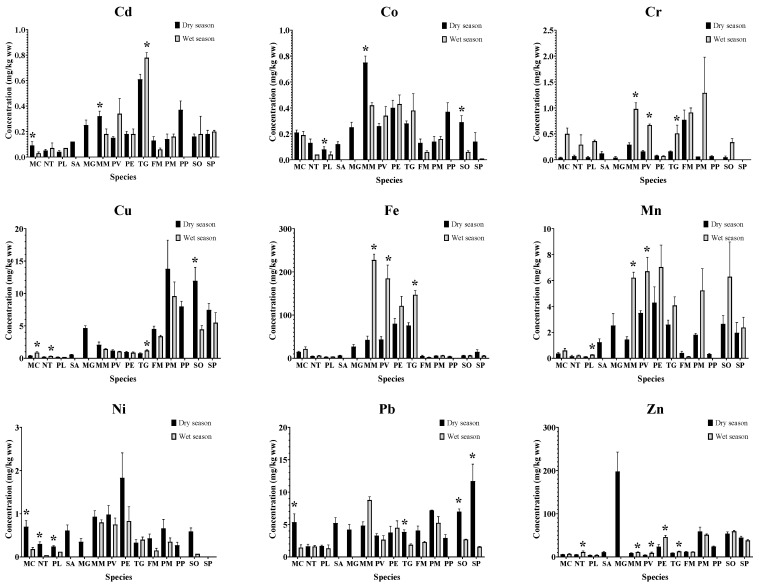
Heavy metal concentrations (mg/kg ww) during the dry and wet seasons in edible tissues of 14 seafood species collected from Pattani Bay during, Pattani province, Thailand. (MC, *Mugil cephalus*; NT, *Netuma thalassina*; PL, *Plotusus lineatus*; SA, *Scatophagus argus*; MG, *Magallana gigas*; MM, *Meretrix meretrix* (L.); PV, *Perna viridis*; PE, *Polymesoda erosa*; TG, *Tegillarca granosa*; FM, *Fenneropenaeus merguiensis* de Man, 1888; PM, *Penaeus monodon*; PP, *Portunus pelagicus*; SO, *Scylla olivacea*; and SP, *Scylla paramamosain*), (* indicates the statistically significant difference of heavy metal concentration between seasons within species with one-way ANOVA analysis at *p* < 0.05).

Average concentrations of nine heavy metals in edible tissues of fish, molluscs, and crustaceans collected from Pattani Bay in wet and dry seasons were shown in Figure 2., The one-way ANOVA analysis showed significant variations of metal concentrations among species indicating that heavy metal accumulation differed in marine tissues. These variations were similar to those found in a previous study [16]. The highest concentrations of most studied heavy metals, except for Cr, Cu, and Pb were found in molluscs and significantly greater than in fish and crustaceans. The greatest concentrations of metals found in molluscs were 0.78 mg Cd/kg ww (*T. granosa*), 0.75 mg Co/kg ww (*M. meretrix*), 227.72 mg Fe/kg ww (*M. meretrix*), 7.04 mg Mn/kg ww (*P. erosa*), 1.83 mg Ni/kg ww (*P. erosa*), and 198.03 mg Zn/kg ww (*M. gigas*). The one-way ANOVA analysis also indicated that crustaceans significantly (*p* < 0.05) accumulated Cu and Pb at the highest concentration of 13.81 mg/kg ww (*P. monodon*), and 11.71 mg/kg ww (*S. paramamosain*), respectively. The differences in metal accumulation among marine species could be related to each species’ unique physiology and ecological niches [39].

The rank order of the average heavy metal accumulation in the edible tissues of all collected seafood species from Pattani Bay was shown in Table 2. The top-ranking essential metal in most species was either Fe or Zn. Iron and Zn are essential trace elements for biota. Molluscs have a high content of Fe as it is a constituent of goethite (α-FeOOH) for the proper functioning of radula [40]. Whereas, Zn is of major importance in metabolic processes: it is a constituent of haemocyanin, hence the level of this element will be higher [40]. Cadmium and Co are toxic elements commonly found at the lowest concentrations. Their levels in seafood species in this study correspond to a previous study and are relevant to the levels found in the environment [6]. Hence the lowest concentrations were observed in animal tissues. Average concentrations of nine heavy metals decreased in the sequence of molluscs > crustaceans > fish. A similar order of heavy metal accumulation was also observed in organisms from other regions, such as Laizhou Bay, China [16,41], and Saint Martin Island, Bangladesh [12]. Marine organisms can directly accumulate heavy metals from water and sediment. There is a strong correlation between heavy metal accumulation in marine species and their habitats, especially if they are in close contact with sediments [42,43,44]. The blood cockle (*T. granosa*), Asiatic hard clam (*M. meretrix*), Asian green mussel (*P. viridis*), common geloina (*P. erosa*), Pacific oyster (*M. gigas*), and giant tiger prawn (*P. monodon*) are species that usually feed on sediment. Their feeding method can contribute to a more significant accumulation of heavy metals than other marine species. Molluscs, especially the filter-feeding animal bivalves, are well-known for their active ingestion of heavy metal-bound organic and inorganic matter. These bivalves can also highly expose to heavy metals during feeding and accumulate a wide range of metals from sediments [45,46]. Moreover, marine organisms can readily assimilate and accumulate the freely dissolved and transported heavy metals in seawater [47]. Among the four studied fish species, *M. cephalus* and *S. argus* are herbivores whereas *N. thalassina* and *P. lineatus* are limnivores or mud-eating. From one-way ANOVA analysis, *S. argus* significantly (*p* < 0.05) accumulated higher concentrations of most heavy metals except Cr and Fe. The variations in heavy metal concentrations among marine species, could thus come from several factors, including feeding strategies, metabolic activities, and rich metal affinity for specific organs [12,16,48,49]. Exposure of various marine species to heavy metals, mainly Cd, Cu, and Zn is associated with the induction of metallothionein. Metallothionein (MT) is a cysteine-rich, low-molecular weight protein that plays a special part in regulating the intracellular homeostasis of essential and non-essential metals, and their detoxification [50,51,52]. Thus, the excess heavy metals will be detoxified by metallothionein and stored in tissues including the liver, kidneys, and muscle. Heavy metal accumulations were observed in the edible tissues of fish (muscle), molluscs (body tissues containing visceral elements), and crustaceans (body and claw tissues) in this study. However, heavy metal concentrations observed in molluscs and crustaceans were higher than in fish. The biomagnification process through the food chain resulted in high levels of heavy metals in higher trophic organisms [53,54,55,56,57,58]. Our findings provide the association between heavy metal accumulation in seafood tissues and their feeding patterns. To avoid health risk effects, consumers should select fish as food instead of molluscs or crustaceans which are more likely to be exposed to heavy metals.

Both national and international permissible limits of heavy metals have been established to ensure food safety and security for human consumption, except Cd, Fe, and Mn [29,59,60]. The concentrations of investigated heavy metals in selected seafood species varied among species (Figure 2). The FAO recommends limits of Cr and Ni for seafood of 12 mg/kg ww and 70 mg/kg ww whereas USFDA set the permissible levels at 13 mg/kg ww and 80 mg/kg ww, respectively [28,30]. Accumulations of Cr and Ni in all seafood species were below both the FAO and USFDA permissible limits [28,30]. The maximum Cd limit in Thailand is 1 mg/kg ww for fish, whereas the limit of CODEX is 2.00 mg/kg ww for bivalve molluscs [25,29]. Cadmium concentrations found in all fish, molluscs, and crustaceans in this study were below both Thailand and CODEX permissible levels. Copper concentrations detected in crustaceans except *F. merguiensis* exceeded the EU limit of 5 mg/kg ww [27]. In contrast, its levels in fish were well below the FAO permissible levels of 30 mg/kg [28]. Copper, an essential element, is easily absorbed by aquatic organisms. This is relevant to the relatively high content in these seafood species [61]. The above limit Cu was also observed in oysters (42.89 mg/kg) [62] and shrimps (5.67 mg/kg) [61]. Copper plays significant roles in the immune, hematopoietic, and cardiovascular systems, and in oxidative stress control [63]. However, excessive Cu can cause gastrointestinal distress and harm the the liver, immune, neurological, and reproductive systems [63]. Iron (Fe) was the most abundant trace element found in all tissue samples ranging from 2.44–227.72 mg/kg ww. Its high concentration in samples indicates that the environment was stressful [64]. Manganese is also an essential element, and its deficiency could lead to severe skeletal and reproductive abnormalities in mammals [65]. Molluscs, in this study, accumulated the highest concentration of Mn (7.04 mg/kg ww; *P. erosa*). Lead, a non-essential element, is well-known for its adverse health effects [60]. Its concentrations in this study ranged from 1.68 to 11.71 mg/kg ww, which were more significant than the maximum EU, FAO, and WHO permitted levels of 1.44, 2.4, and 9.6 mg/kg ww, respectively [26,28,31]. In addition, high concentrations of Pb were recorded from the topsoil of the Pattani River mouth (557.15 mg/kg), resulting in high Pb contamination in Pattani Bay (6.43–69.49 mg/kg). This can increase the risk to aquatic animals and local human health [6]. Present findings showed that Pb concentrations in detected seafood species were relatively high for human consumption. Acute exposure to high Pb levels can cause gastrointestinal, renal, and brain damage along with other toxic effects [66]. Zinc, an element essential for metabolic processes, was also found in all samples. Most Zn concentrations in all species were below the permissible of 100 and 1000 mg/kg ww set by WHO, for fish and crustaceans, respectively [67,68], except for *M. gigas* (198 mg/kg ww).

**Figure 3:**
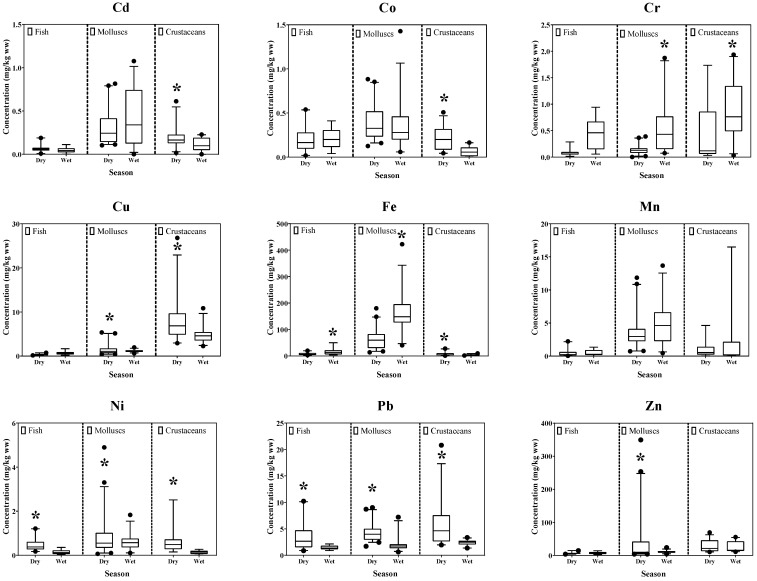
Nine heavy metal accumulations in fish, molluscs, and crustaceans were collected during dry and wet seasons from Pattani Bay, Pattani province, Thailand. (• indicates the outliers, * indicates the statistically significant difference of heavy metal concentration between seasons within the same organism type using the paired sample *t*-test between dry and wet seasons).

Average heavy metal accumulation in fish, molluscs, and crustaceans is shown in Figure 3. The paired sample *t*-test indicated that seasonal and collecting location variations affected heavy metal concentrations in seafood tissues. Seasonal variations affected heavy metal accumulation for most studied heavy metals except Mn. Fish showed significantly higher concentrations (*p* < 0.05) of Ni and Pb in the dry season and of Cr and Fe in the wet season. Molluscs showed substantially higher levels (*p* < 0.05) of Cu, Pb, and Zn in the dry season, and Cr and Fe in the wet season. Crustaceans showed significantly greater levels (*p* < 0.05) of Cd, Co, Cu, Fe, Ni, and Pb in the dry season, and Cr in the wet season. Levels of most heavy metals in the dry season (summer) were relatively higher than in the wet season. This could be attributed to a higher influx of agricultural waste, sewage, and sludge by heavy rainfall and flooding [60].

Seasonal fluctuations of heavy metals can result from several factors, such as growth, organismal reproductive cycles, and water temperature changes. These factors could contribute additional factors that affect metal bioavailability in marine organismal tissues [69,70,71]. Studies linked to the reproductive cycle can explain the essential seasonal variations of heavy metal bioaccumulation in mollusc tissues [70,71]. Marine animal gonads are increased enormously during gametogenesis and could constitute an effective trap for incorporating metals into living organisms. This is related to cellular energy mainly used in gamete production [59]. Our results showed high Cu, Pb, and Zn concentrations in molluscs during the dry season. This coincided with gametogenesis processes taking place during such a period. The present study also found a higher concentration of Ni and Pb in fish tissues and Cd, Co, Cu, Fe, Ni, and Pb in crustacean tissues during the dry season, similar to other studies [72,73,74]. Fish and shrimps increase their physiological motion in the dry season which could produce a higher accumulation of heavy metals [75]. The growth rate of fish is higher in summer, and can thus result in greater heavy metal accumulation [76]. The gradual accumulation of nutrients during the pre-spawning season in dry season could introduce significant variations of metal concentrations and metallothionein content in the digestive gland due to the “biological dilution” effect [77]. Therefore, these can also increase heavy metal accumulation in tissues during the dry season. Our findings provide evidence that most heavy metals accumulated in seafood tissues during the dry season were higher than in the wet season. This finding would suggest that seafood consumption during the wet season would cause lower health risk effects than in the dry season. The monitoring of heavy metals contaminated seafood should periodically be performed to prevent health risks of consumers, especially those in the surrounding areas.

### 3.2. Relationship Amongst Heavy Metal Concentrations in Seafood

**Figure 4:**
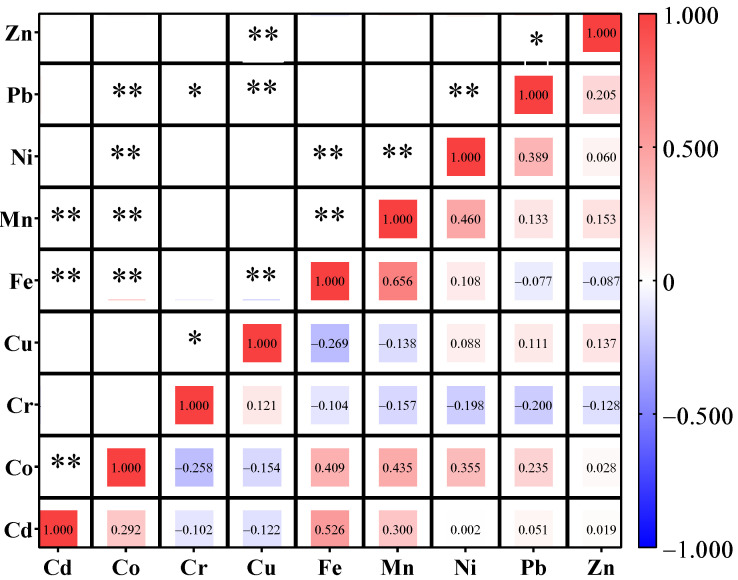
Pearson correlation analysis of heavy metal accumulations in 14 seafood species collected from Pattani Bay, Pattani province, Thailand. (* Correlation is significant at the 0.05 level ** Correlation is significant at the 0.01 level).

Pearson correlation analysis of metal concentrations in edible tissues of seafood organisms collected in Pattani Bay, Thailand, were presented in Figure 4. These correlations reflected similar sources of pollution or similar cumulative characteristics among species of the corresponding samples [16]. Fe was positively and significantly (*p* < 0.01) correlated with most heavy metals except Cr, Cu, and Zn. This indicated that the accumulation of Fe in the edible tissue of studied species was closely related to those heavy metals. Similar accumulative characteristics also occurred with other heavy metal groups [16]. These could be due to different pollution sources and marine tissue variations [78,79,80,81].

**Figure 5:**
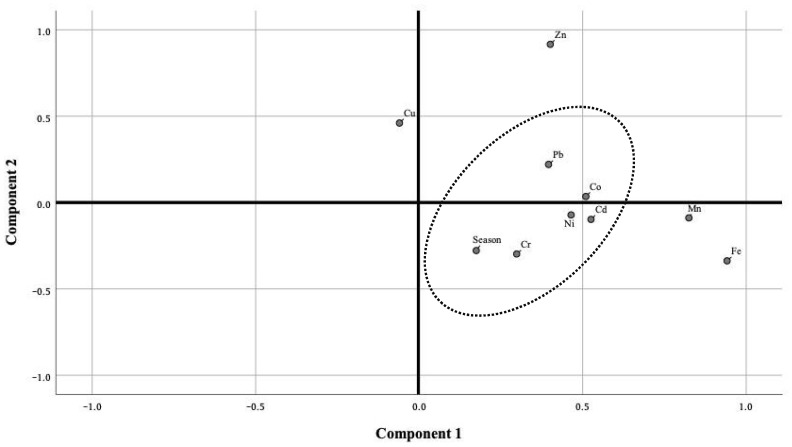
Rotated component loading plot for nine heavy metals in seafood collected from Pattani Bay, Pattani province, Thailand.

The PCA used to identify the associations among nine heavy metals in seafood tissues and seasons is shown in Figure 5. The rotated component loading plot revealed that the first two principal components of the PCA accounted for 99.84% of the total variance. The first and second principal components accounted for 65.82% and 32.02% of the total variance, respectively. However, heavy metal relationships were close within the first compartment by season and toxic heavy metals (Cd, Co, Cr, Ni, and Pb). This identified that these toxic heavy metals showed similar accumulative characteristics in their tissues. These are likely due to different pollution sources and marine tissue variations [78,79,80,81].

### 3.3. Human Health Risk Assessment of Heavy Metal Concentration via Seafood Consumption

#### 3.3.1. Non-Carcinogenic Human Health Risk

The THQ used to assess non-carcinogenic human health risks from seafood consumption of nine heavy metals from Pattani Bay was calculated and presented in Table 3. The acceptable THQ value is ≤1 [36]. The THQ and HI values of all heavy metals in studied seafood species were below 1. The highest THQ value was found in *P. monodon* (8.97 × 10^−3^) for Cr, whereas the highest HI value was observed in a *P. monodon* (1.55 × 10^−2^).

The THQ value is a reasonable parameter for assessing human health risks from heavy metal contaminated seafood consumption [82,83]. The population exposed to heavy metals via contaminated food consumption with a THQ value above 1 could have deleterious effects [12]. Humans exposed to more than one pollutant may suffer combined or interactive effects [84]. This present study indicated that the average consumption of mixed heavy metal-contaminated seafood is unlikely to pose significant risks to the human health of both Thai males and females. However, it should be noted that excessive consumption of these seafood in coastal areas should be avoided to prevent adverse health effects from multiple heavy metal exposure.

#### 3.3.2. Carcinogenic Human Health Risk

The TR values used to determine carcinogenic human health risks from the consumption of seafood collected from Pattani Bay were presented in Table 4. The carcinogenic potencies of the oral slope factor are available for Cr, Ni, and Pb in Table 1 [33,85]. Cancer health risk above 10^−4^ is unacceptable, between 10^−6^ to 10^−4^ is acceptable, and below 10^−6^ is negligible [36]. This study found that most carcinogenic risks were negligible except for Cr. The TR of Cr was acceptable if *P. monodon* were consumed. Chromium is an essential trace element and plays a beneficial role in insulin molecules to bring glucose into cells for glycolysis. That is the first step in ATP production and lipid metabolism in organisms [86,87]. Chromium compounds are carcinogenic in a variety of test systems in animals. Long-term Cr exposure can cause damage to the nose, skin, lungs, and stomach, as well as convulsions and even death [88]. The levels of all heavy metal accumulation in seafood did not exceed the acceptable TR range. This indicated that consuming heavy metal-contaminated seafood from Pattani Bay would not cause human carcinogenic risk. However, contamination and accumulation studies of heavy metals should periodically be performed to monitor the possible human health risk from the consumption of seafood species.

## 4. Conclusions

The present study highlighted human health risks based on heavy metal exposure following the consumption of polluted seafood in Pattani Bay, Thailand. The significant differences in heavy metal levels in selected seafood species were observed depending on both seasons and collected locations. All collected 14 seafood species from Pattani Bay were safe for consumption. There are no possibilities of both non-carcinogenic and carcinogenic health risks associated with continuous consumption for 70 years. However, uncontrolled releases of toxic heavy metals from human activities can increase heavy metal levels in seafood. The contamination and accumulation studies of heavy metals, therefore, should periodically be performed to monitor possible human health risks from seafood consumption.

## Figures and Tables

**Table 1:** Oral reference dose (RfD) and oral cancer slope factor of nine heavy metals used for human health risk assessment.

Parameter	Value								
	Cd	Co	Cr	Cu	Fe	Mn	Ni	Pb	Zn
Oral Reference Dose (RfD) (mg/kg-day)	0.001 *	0.03 **	0.003 *	0.5 ***	0.8 ***	0.14 *	0.02 *	0.025 ***	0.3 *
Oral Cancer Slope Factor (per mg/kg-day)	-	-	0.5 *****	-	-	-	0.91 ****	8.50 × 10^−3^ ****	-

* IRIS [35] ** Finley, Monnot, Paustenbach and Gaffney [34] *** WHO [31] **** CalEPA [33] ***** USEPA [36].

**Table 2:** Rank order of heavy metal accumulation in edible tissues of 14 seafood species collected from Pattani Bay, Pattani province, Thailand.

Group	Species	Order
Fish	*Mugil cephalus*	Fe > Zn > Pb > Cu > Mn > Ni > Cr > Co > Cd
	*Netuma thalassina*	Zn > Fe > Pb > Cu > Ni > Mn > Cr > Co > Cd
	*Plotosus lineatus*	Zn > Fe > Pb > Ni > Cu > Mn > Co > Cr > Cd
	*Scatophagus argus*	Zn > Fe > Pb > Mn > Ni > Cu > Co > Cr > Cd
Molluscs	*Magallana gigas*	Zn > Fe > Cu > Pb > Mn > Ni > Co > Cd > Cr
	*Meretrix meretrix* (L.)	Fe > Zn > Mn > Pb > Cu > Ni > Cr > Co > Cd
	*Perna viridis*	Fe > Zn > Mn > Pb > Cu > Ni > Co > Cr > Cd
	*Polymesoda erosa*	Fe > Zn > Mn > Pb > Ni > Cu > Co > Cd > Cr
	*Tegillarca granosa*	Fe > Zn > Mn > Pb > Cu > Ni > Cr > Co > Cd
Crustaceans	*Fenneropenaeus merguiensis* de Man, 1888	Zn > Fe > Cu > Pb > Cr > Mn > Ni > Co > Cd
	*Penaeus monodon*	Zn > Cu > Fe > Pb > Cr > Mn > Ni > Co > Cd
	*Portunus pelagicus*	Zn > Cu > Fe > Pb > Cd > Mn > Ni > Co > Cr
	*Scylla olivacea*	Zn > Cu > Fe > Mn > Pb > Ni > Cr > Co > Cd
	*Scylla paramamosain*	Zn > Fe > Pb > Cu > Mn > Ni > Cr > Cd > Co

**Table 3:** Target hazard quotient (THQ) and hazard index (HI) of edible tissues of 14 seafood species by Thai male and female adults.

Species Name	Sex	Target Hazard Quotient (THQ)									Hazard Index (HI)
		Cd	Co	Cr	Cu	Fe	Mn	Ni	Pb	Zn	
Fish											
*Mugil* *cephalus*	Male	2.26 × 10^−5^	2.75 × 10^−6^	5.75 × 10^−5^	5.85 × 10^−7^	9.94 × 10^−6^	1.55 × 10^−6^	7.26 × 10^−6^	4.58 × 10^−5^	9.14 × 10^−6^	1.57 × 10^−4^
	Female	2.49 × 10^−5^	3.04 × 10^−6^	6.34 × 10^−5^	6.45 × 10^−7^	1.10 × 10^−5^	1.71 × 10^−6^	8.00 × 10^−6^	5.05 × 10^−5^	1.01 × 10^−5^	1.73 × 10^−4^
*Netuma* *thalassina*	Male	1.14 × 10^−5^	7.82 × 10^−7^	9.02 × 10^−6^	1.05 × 10^−7^	1.32 × 10^−6^	2.54 × 10^−7^	2.25 × 10^−6^	1.31 × 10^−5^	4.83 × 10^−6^	4.30 × 10^−5^
	Female	1.25 × 10^−5^	8.63 × 10^−7^	9.94 × 10^−6^	1.16 × 10^−7^	1.45 × 10^−6^	2.80 × 10^−7^	2.48 × 10^−6^	1.44 × 10^−5^	5.32 × 10^−6^	4.74 × 10^−5^
*Plotosus* *lineatus*	Male	9.72 × 10^−6^	1.14 × 10^−6^	9.99 × 10^−6^	7.79 × 10^−8^	8.56 × 10^−7^	2.44 × 10^−7^	2.22 × 10^−6^	1.27 × 10^−5^	2.91 × 10^−6^	3.99 × 10^−5^
	Female	1.07 × 10^−5^	1.25 × 10^−6^	1.10 × 10^−5^	8.59 × 10^−8^	9.44 × 10^−7^	2.70 × 10^−7^	2.45 × 10^−6^	1.40 × 10^−5^	3.21 × 10^−6^	4.40 × 10^−5^
*Scatophagus* *argus*	Male	6.67 × 10^−4^	7.51 × 10^−5^	2.39 × 10^−4^	6.37 × 10^−6^	4.66 × 10^−5^	5.06 × 10^−5^	1.77 × 10^−4^	1.21 × 10^−3^	2.12 × 10^−4^	2.68 × 10^−3^
	Female	7.36 × 10^−4^	8.28 × 10^−5^	2.64 × 10^−4^	7.02 × 10^−6^	5.14 × 10^−5^	5.58 × 10^−5^	1.96 × 10^−4^	1.33 × 10^−3^	2.33 × 10^−4^	2.96 × 10^−3^
Molluscs											
*Magallana* *gigas*	Male	1.04 × 10^−3^	3.66 × 10^−5^	7.44 × 10^−5^	3.89 × 10^−5^	1.41 × 10^−4^	7.53 × 10^−5^	7.30 × 10^−5^	7.04 × 10^−4^	2.75 × 10^−3^	4.94 × 10^−3^
	Female	1.15 × 10^−3^	4.04 × 10^−5^	8.20 × 10^−5^	4.29 × 10^−5^	1.55 × 10^−4^	8.30 × 10^−5^	8.05 × 10^−5^	7.76 × 10^−4^	3.04 × 10^−3^	5.45 × 10^−3^
*Meretrix* *meretrix* (L.)	Male	1.58 × 10^−3^	1.24 × 10^−4^	1.35 × 10^−3^	2.22 × 10^−5^	1.07 × 10^−3^	1.73 × 10^−4^	2.74 × 10^−4^	9.51 × 10^−4^	2.08 × 10^−4^	5.75 × 10^−3^
	Female	1.74 × 10^−3^	1.36 × 10^−4^	1.48 × 10^−3^	2.45 × 10^−5^	1.18 × 10^−3^	1.91 × 10^−4^	3.02 × 10^−4^	1.05 × 10^−3^	2.30 × 10^−4^	6.34 × 10^−3^
*Perna* *viridis*	Male	4.55 × 10^−5^	1.83 × 10^−6^	1.53 × 10^−5^	4.28 × 10^−7^	2.75 × 10^−5^	6.92 × 10^−6^	7.83 × 10^−6^	2.20 × 10^−5^	4.34 × 10^−6^	1.32 × 10^−4^
	Female	5.01 × 10^−5^	2.02 × 10^−6^	1.68 × 10^−5^	4.72 × 10^−7^	3.04 × 10^−5^	7.64 × 10^−6^	8.64 × 10^−6^	2.42 × 10^−5^	4.79 × 10^−6^	1.45 × 10^−4^
*Polymesoda erosa*	Male	3.29 × 10^−5^	2.54 × 10^−6^	4.69 × 10^−6^	3.48 × 10^−7^	2.18 × 10^−5^	6.92 × 10^−6^	1.35 × 10^−5^	2.99 × 10^−5^	1.93 × 10^−5^	1.32 × 10^−4^
	Female	3.63 × 10^−5^	2.80 × 10^−6^	5.17 × 10^−6^	3.84 × 10^−7^	2.40 × 10^−5^	7.63 × 10^−6^	1.48 × 10^−5^	3.30 × 10^−5^	2.13 × 10^−5^	1.45 × 10^−4^
*Tegillarca granosa*	Male	1.29 × 10^−4^	2.07 × 10^−6^	2.08 × 10^−5^	3.64 × 10^−7^	2.59 × 10^−5^	4.44 × 10^−6^	3.41 × 10^−6^	2.13 × 10^−5^	6.83 × 10^−6^	2.15 × 10^−4^
	Female	1.43 × 10^−4^	2.28 × 10^−6^	2.29 × 10^−5^	4.01 × 10^−7^	2.86 × 10^−5^	4.90 × 10^−6^	3.76 × 10^−6^	2.35 × 10^−5^	7.53 × 10^−6^	2.37 × 10^−4^
Crustaceans											
*Fenneropenaeus merguiensis* de Man, 1888	Male	2.21 × 10^−3^	9.77 × 10^−5^	5.70 × 10^−3^	1.73 × 10^−4^	1.12 × 10^−4^	4.79 × 10^−5^	3.21 × 10^−4^	2.92 × 10^−3^	8.34 × 10^−4^	1.24 × 10^−2^
	Female	2.44 × 10^−3^	1.08 × 10^−4^	6.29 × 10^−3^	1.91 × 10^−4^	1.24 × 10^−4^	5.29 × 10^−5^	3.53 × 10^−4^	3.21 × 10^−3^	9.20 × 10^−4^	1.37 × 10^−2^
*Penaeus* *monodon*	Male	1.65 × 10^−3^	8.45 × 10^−5^	8.13 × 10^−3^	3.21 × 10^−4^	8.08 × 10^−5^	3.51 × 10^−5^	1.93 × 10^−4^	2.48 × 10^−3^	1.08 × 10^−3^	1.41 × 10^−2^
	Female	1.82 × 10^−3^	9.31 × 10^−5^	8.97 × 10^−3^	3.53 × 10^−4^	8.91 × 10^−5^	3.87 × 10^−5^	2.13 × 10^−4^	2.73 × 10^−3^	1.19 × 10^−3^	1.55 × 10^−2^
*Portunus* *pelagicus*	Male	1.36 × 10^−3^	3.22 × 10^−5^	8.64 × 10^−5^	5.81 × 10^−5^	1.88 × 10^−5^	8.26 × 10^−6^	4.99 × 10^−5^	4.25 × 10^−5^	2.90 × 10^−4^	2.33 × 10^−3^
	Female	1.50 × 10^−3^	3.56 × 10^−5^	9.53 × 10^−5^	6.41 × 10^−5^	2.07 × 10^−5^	9.11 × 10^−9^	5.50 × 10^−5^	4.68 × 10^−4^	3.20 × 10^−4^	2.56 × 10^−3^
*Scylla* *olivacea*	Male	6.19 × 10^−4^	2.69 × 10^−5^	3.09 × 10^−4^	5.97 × 10^−5^	2.80 × 10^−5^	1.42 × 10^−4^	9.11 × 10^−5^	6.90 × 10^−4^	6.27 × 10^−4^	2.59 × 10^−3^
	Female	6.83 × 10^−4^	2.96 × 10^−5^	3.41 × 10^−4^	6.59 × 10^−5^	3.08 × 10^−5^	1.57 × 10^−4^	1.01 × 10^−4^	7.61 × 10^−4^	6.91 × 10^−4^	2.86 × 10^−3^
*Scylla* *paramamosain*	Male	6.88 × 10^−4^	9.00 × 10^−6^	5.45 × 10^−4^	4.71 × 10^−5^	4.65 × 10^−5^	5.64 × 10^−5^	2.84 × 10^−4^	9.67 × 10^−4^	5.01 × 10^−4^	3.14 × 10^−3^
	Female	7.59 × 10^−4^	9.92 × 10^−6^	6.01 × 10^−4^	5.19 × 10^−5^	5.12 × 10^−5^	6.22 × 10^−5^	3.13 × 10^−4^	1.07 × 10^−3^	5.53 × 10^−4^	3.47 × 10^−3^

**Table 4:** Target cancer risk (TR) of selected toxic heavy metals from the consumption of edible tissues of 14 seafood species by Thai male and female adults.

Group	Species Name	Target Cancer Risk (TR)					
		Cr		Ni		Pb	
		Male	Female	Male	Female	Male	Female
Fish	*Mugil cephalus*	8.63 × 10^−8^	9.51 × 10^−8^	1.32 × 10^−7^	1.46 × 10^−7^	9.72 × 10^−9^	1.07 × 10^−8^
	*Netuma thalassina*	1.35 × 10^−8^	1.49 × 10^−8^	4.10 × 10^−8^	4.52 × 10^−8^	2.78 × 10^−9^	3.07 × 10^−9^
	*Plotosus lineatus*	1.50 × 10^−8^	1.65 × 10^−8^	4.04 × 10^−8^	4.46 × 10^−8^	2.70 × 10^−9^	2.98 × 10^−9^
	*Scatophagus argus*	3.59 × 10^−7^	3.96 × 10^−7^	3.23 × 10^−6^	3.56 × 10^−6^	2.57 × 10^−7^	2.83 × 10^−7^
Molluscs	*Magallana gigas*	1.12 × 10^−7^	1.23 × 10^−7^	1.33 × 10^−6^	1.47 × 10^−6^	1.50 × 10^−7^	1.65 × 10^−7^
	*Meretrix meretrix* (L.)	2.02 × 10^−6^	2.22 × 10^−6^	4.99 × 10^−6^	5.50 × 10^−6^	2.02 × 10^−7^	2.23 × 10^−7^
	*Perna viridis*	2.29 × 10^−8^	2.53 × 10^−8^	1.43 × 10^−7^	1.57 × 10^−7^	4.67 × 10^−9^	5.15 × 10^−9^
	*Polymesoda erosa*	7.04 × 10^−9^	7.76 × 10^−9^	2.45 × 10^−7^	2.70 × 10^−7^	6.36 × 10^−9^	7.01 × 10^−9^
	*Tegillarca granosa*	3.11 × 10^−8^	3.43 × 10^−8^	6.21 × 10^−8^	6.84 × 10^−8^	4.52 × 10^−9^	4.98 × 10^−9^
Crustaceans	*Fenneropenaeus merguiensis* de Man, 1888	8.55 × 10^−6^	9.43 × 10^−6^	5.84 × 10^−6^	6.43 × 10^−6^	6.19 × 10^−7^	6.83 × 10^−7^
	*Penaeus monodon*	1.22 × 10^−5^	1.34 × 10^−5^	3.52 × 10^−6^	3.88 × 10^−6^	5.26 × 10^−7^	5.80 × 10^−7^
	*Portunus pelagicus*	1.30 × 10^−7^	1.43 × 10^−7^	9.08 × 10^−7^	1.00 × 10^−6^	9.03 × 10^−8^	9.95 × 10^−8^
	*Scylla olivacea*	4.63 × 10^−7^	5.11 × 10^−7^	1.66 × 10^−6^	1.83 × 10^−6^	1.47 × 10^−7^	1.62 × 10^−7^
	*Scylla paramamosain*	8.18 × 10^−7^	9.01 × 10^−7^	5.17 × 10^−6^	5.70 × 10^−6^	2.05 × 10^−7^	2.27 × 10^−7^

## Data Availability

Not applicable.

## References

[1] Rajaram R., Ganeshkumar A., Vinothkannan A. (2020). Health risk assessment and bioaccumulation of toxic metals in commercially important finfish and shellfish resources collected from Tuticorin coast of Gulf of Mannar, Southeastern India. Mar. Pollut. Bull..

[2] Wang X., Zhao L., Xu H., Zhang X. (2018). Spatial and seasonal characteristics of dissolved heavy metals in the surface seawater of the Yellow River Estuary, China. Mar. Pollut. Bull..

[3] Burgos-Núñez S., Navarro-Frómeta A., Marrugo-Negrete J., Enamorado-Montes G., Urango-Cárdenas I. (2017). Polycyclic aromatic hydrocarbons and heavy metals in the Cispata Bay, Colombia: A marine tropical ecosystem. Mar. Pollut. Bull..

[4] Suwanjarat J., Pituksalee C., Thongchai S. (2009). Reproductive cycle of *Anadara granosa* at Pattani Bay and its relationship with metal concentrations in the sediments. Songklanakarin J. Sci. Technol..

[5] Wang K., Shi X., Qiao S., Kornkanitnan N., Khokiattiwong S. (2015). Distribution and composition of authigenic minerals in surface sediments of the western Gulf of Thailand. Acta Oceanol. Sin..

[6] Sowana A., Shrestha R.P., Parkpian P., Pongquan S. (2011). Influence of coastal land use on soil heavy-metal contamination in Pattani Bay, Thailand. J. Coast. Res..

[7] Prato E., Biandolino F., Parlapiano I., Giandomenico S., Denti G., Calò M., Spada L., Di Leo A. (2019). Proximate, fatty acids and metals in edible marine bivalves from Italian market: Beneficial and risk for consumers health. Sci. Total Environ..

[8] McManus A., Newton W. (2011). Seafood, Nutrition and Human Health: A Synopsis of the Nutritional Benefits of Consuming Seafood.

[9] Sacchettini G., Castellini G., Graffigna G., Hung Y., Lambri M., Marques A., Perrella F., Savarese M., Verbeke W., Capri E. (2021). Assessing consumers’ attitudes, expectations and intentions towards health and sustainability regarding seafood consumption in Italy. Sci. Total Environ..

[10] Pandion K., Khalith S.B.M., Ravindran B., Chandrasekaran M., Rajagopal R., Alfarhan A., Chang S.W., Ayyamperumal R., Mukherjee A., Arunachalam K.D. (2022). Potential health risk caused by heavy metal associated with seafood consumption around coastal area. Environ. Pollut..

[11] Zhao B., Wang X., Jin H., Feng H., Shen G., Cao Y., Yu C., Lu Z., Zhang Q. (2018). Spatiotemporal variation and potential risks of seven heavy metals in seawater, sediment, and seafood in Xiangshan Bay, China (2011–2016). Chemosphere.

[12] Baki M.A., Hossain M.M., Akter J., Quraishi S.B., Haque Shojib M.F., Atique Ullah A.K.M., Khan M.F. (2018). Concentration of heavy metals in seafood (fishes, shrimp, lobster and crabs) and human health assessment in Saint Martin Island, Bangladesh. Ecotoxicol. Environ. Saf..

[13] Aranda N., Valls R.M., Romeu M., Sánchez-Martos V., Albaladejo R., Fernández-Castillejo S., Nogués R., Catalán Ú., Pedret A., Espinel A. (2017). Consumption of seafood and its estimated heavy metals are associated with lipid profile and oxidative lipid damage on healthy adults from a Spanish Mediterranean area: A cross-sectional study. Environ. Res..

[14] WHO (2006). Elemental Speciation in Human Health Risk Assessment.

[15] Tongesayi T., Fedick P., Lechner L., Brock C., Le Beau A., Bray C. (2013). Daily bioaccessible levels of selected essential but toxic heavy metals from the consumption of non-dietary food sources. Food Chem. Toxicol..

[16] Liu Q., Liao Y., Shou L. (2018). Concentration and potential health risk of heavy metals in seafoods collected from Sanmen Bay and its adjacent areas, China. Mar. Pollut. Bull..

[17] Ulaganathan A., Robinson J.S., Rajendran S., Geevaretnam J., Pandurangan P., Durairaj S. (2022). Effect of different thermal processing methods on potentially toxic metals in the seafood, *Penaeus vannamei*, and the related human health risk assessment. J. Food Compos. Anal..

[18] Bao C., Cai Q., Ying X., Zhu Y., Ding Y., Murk T.A.J. (2021). Health risk assessment of arsenic and some heavy metals in the edible crab (*Portunus trituberculatus*) collected from Hangzhou Bay, China. Mar. Pollut. Bull..

[19] Carpenter K.E., Niem V.H. (1998). FAO Species Identification Guide for Fishery Purposes. The Living Marine Resources of the Western Central Pacific Volume 2: Cephalopods, Crustaceans, Holothurians and Sharks.

[20] Carpenter K.E., Niem V.H. (1998). FAO Species Identification Guide for Fishery Purposes. The Living Marine Resources of the Western Central Pacific: Volume 1. Seaweeds, Corals, Bivalves and Gastropods.

[21] Carpenter K.E., Niem V.H. (1999). FAO Species Identification Guide for Fishery Purposes. The Living Marine Resources of the Western Central Pacific: Volume 3. Batoid Fishes, Chimaeras and Bony Fishes Part 1.

[22] Carpenter K.E., Niem V.H. (1999). FAO Species Identification Guide for Fishery Purposes. The Living Marine Resources of the Western Central Pacific: Volume 4. Bony Fishes Part 2 (Mugilidae to Carangidae).

[23] Carpenter K.E., Niem V.H. (2001). FAO Species Identification Guide for Fishery Purposes. The Living Marine Resources of the Western Central Pacific: Volume 5. Bony Fishes Part 3 (Menidae to Pomacentridae).

[24] Carpenter K.E., Niem V.H. (2001). FAO Species Identification Guide for Fishery Purposes. The Living Marine Resources of the Western Central Pacific: Volume 6. Bony Fishes Part 4 (Labridae to Latimeriidae), Estuarine Crocodiles, Sea Turtles, Sea Snakes and Marine Mammals.

[25] CodexAlimentarius (1995). Codex General Standard for Contaminants and Toxins in Foods. Codex Standard 193-1995.

[26] EU (2001). Commission Regulation (EC). No 466/2001 of 8 March 2001 Setting Maximum Levels for Certain Contaminants in Foodstuffs.

[27] EU (2006). Commission Regulation (EC). No. 1881/2006 of 19 December Setting Maximum Levels for Certain Contaminants in Foodstuffs.

[28] FAO (1983). Compilation of Legal Limits for Hazardous Substances in Fish and Fishery Products. FAO Fishery Circular.

[29] Ministry of Public Health Thailand (2020). Standards for Contaminants in Food.

[30] USFDA (1993). Food and Drug Administration.

[31] WHO Evaluation of Joint FAO/WHO Expert Committee on Food Additives (JECFA). www.apps.who.int/food-additives-contaminants-jecfa-database.

[32] Rakib M.R.J., Jolly Y.N., Enyoh C.E., Khandaker M.U., Hossain M.B., Akther S., Alsubaie A., Almalki A.S.A., Bradley D.A. (2021). Levels and health risk assessment of heavy metals in dried fish consumed in Bangladesh. Sci. Rep..

[33] CalEPA Chemicals Known to the State to Cause Cancer or Reproductive Toxicity. https://oehha.ca.gov/proposition-65/proposition-65-list.

[34] Finley B.L., Monnot A.D., Paustenbach D.J., Gaffney S.H. (2012). Derivation of a chronic oral reference dose for cobalt. Regul. Toxicol. Pharmacol..

[35] IRIS Integrated Risk Informaion System. http://cfpub.epa.gov/ncea/iris/index.cfm.

[36] USEPA (2000). Risk-Based Concentration Table.

[37] WHO GHE: Life Expectancy and Healthy Life Expectancy. https://www.who.int/data/gho/data/indicators/indicator-details/GHO/gho-ghe-life-tables-by-country.

[38] USEPA (2011). Exposure Factor Hanbook.

[39] Gholamhosseini A., Shiry N., Soltanian S., Banaee M. (2021). Bioaccumulation of metals in marine fish species captured from the northern shores of the Gulf of Oman, Iran. Reg. Stud. Mar. Sci..

[40] Anna J., Piotr K., Krzysztof S., Jacek N. (2011). Bioaccumulation of Metals in Tissues of Marine Animals, Part II: Metal Concentrations in Animal Tissues. Pol. J. Environ. Stud..

[41] Liu H., Liu G., Yuan Z., Ge M., Wang S., Liu Y., Da C. (2019). Occurrence, potential health risk of heavy metals in aquatic organisms from Laizhou Bay, China. Mar. Pollut. Bull..

[42] Ravanbakhsh M., Zare Javid A., Hadi M., Jaafarzadeh Haghighi Fard N. (2020). Heavy metals risk assessment in fish species (*Johnius belangerii* (C) and *Cynoglossus arel*) in Musa Estuary, Persian Gulf. Environ. Res..

[43] Janadeleh H., Jahangiri S. (2016). Risk assessment and heavy metal contamination in fish (*Otolithes ruber*) and sediments in Persian Gulf. J. Community Health Res..

[44] Ahmed Q., Benzer S., Ali Q.M. (2018). Heavy metal concentration in largehead hairtail (*Trichiurus lepturus* Linneaus, 1758) and Savalai hairtail (*Lepturacanthus savala* (Cuvier, 1829)) obtained from Karachi fish harbour, Pakistan. Bull. Environ. Contam. Toxicol..

[45] Bartolomé L., Navarro P., Raposo J.C., Arana G., Zuloaga O., Etxebarria N., Soto M. (2010). Occurrence and distribution of metals in mussels from the Cantabrian Coast. Arch. Environ. Contam. Toxicol..

[46] Fernández B., Campillo J.A., Martínez-Gómez C., Benedicto J. (2010). Antioxidant responses in gills of mussel (*Mytilus galloprovincialis*) as biomarkers of environmental stress along the Spanish Mediterranean coast. Aquat. Toxicol..

[47] Jordão C.P., Pereira M.G., Bellato C.R., Pereira J.L., Matos A.T. (2002). Assessment of water systems for contaminants from domestic and industrial sewages. Environ. Monit. Assess..

[48] Liu J., Cao L., Dou S. (2017). Bioaccumulation of heavy metals and health risk assessment in three benthic bivalves along the coast of Laizhou Bay, China. Mar. Pollut. Bull..

[49] Sarkar T., Alam M.M., Parvin N., Fardous Z., Chowdhury A.Z., Hossain S., Haque M.E., Biswas N. (2016). Assessment of heavy metals contamination and human health risk in shrimp collected from different farms and rivers at Khulna-Satkhira region, Bangladesh. Toxicol. Rep..

[50] Amiard J.C., Amiard-Triquet C., Barka S., Pellerin J., Rainbow P.S. (2006). Metallothioneins in aquatic invertebrates: Their role in metal detoxification and their use as biomarkers. Aquat. Toxicol..

[51] Roesijadi G. (1992). Metallothioneins in metal regulation and toxicity in aquatic animals. Aquat. Toxicol..

[52] Viarengo A., Nott J.A. (1993). Mechanisms of heavy metal cation homeostasis in marine invertebrates. Comp. Biochem. Physiol. Part C Comp. Pharmacol..

[53] Christopher B.N., Ekaluo U.B., Asuquo F.E. (2010). Comparative bioaccumulation of heavy metals (Fe, Mn, Zn, Cu, Cd & Cr) by some edible aquatic mollusc from the Atlantic Coastline of Soulth Eastern Nigeria. World J. Fish Mar. Sci..

[54] El-Kady A.A., Abdel-Wahhab M.A. (2018). Occurrence of trace metals in foodstuffs and their health impact. Trends Food Sci. Technol..

[55] Hao Z., Chen L., Wang C., Zou X., Zheng F., Feng W., Zhang D., Peng L. (2019). Heavy metal distribution and bioaccumulation ability in marine organisms from coastal regions of Hainan and Zhoushan, China. Chemosphere.

[56] Liao C.M., Ling M.P. (2003). Assessment of human health risks for arsenic bioaccumulation in tilapia (*Oreochromis mossambicus*) and large-scale mullet (*Liza macrolepis*) from blackfoot disease area in Taiwan. Arch. Environ. Contam. Toxicol..

[57] Malik N., Biswas A.K., Qureshi T.A., Borana K., Virha R. (2010). Bioaccumulation of heavy metals in fish tissues of a freshwater lake of Bhopal. Environ. Monit. Assess..

[58] Yusoff N.A.M., Long S.M. Comparative bioaccumulation of heavy metals (Fe, Zn, Cu, Cd, Cr, Pb) in different edible mollusk collected from the estuary area of Sarawak river. Proceedings of the Universiti Malaysia Terengganu 10th International Annual Symposium (UMTAS 2011).

[59] Azizi G., Layachi M., Akodad M., Yáñez-Ruiz D.R., Martín-García A.I., Baghour M., Mesfioui A., Skalli A., Moumen A. (2018). Seasonal variations of heavy metals content in mussels (*Mytilus galloprovincialis*) from Cala Iris offshore (Northern Morocco). Mar. Pollut. Bull..

[60] Saha N., Mollah M.Z.I., Alam M.F., Safiur Rahman M. (2016). Seasonal investigation of heavy metals in marine fishes captured from the Bay of Bengal and the implications for human health risk assessment. Food Control.

[61] Yu B., Wang X., Dong K.F., Xiao G., Ma D. (2020). Heavy metal concentrations in aquatic organisms (fishes, shrimp and crabs) and health risk assessment in China. Mar. Pollut. Bull..

[62] Liu Q., Xu X., Zeng J., Shi X., Liao Y., Du P., Tang Y., Huang W., Chen Q., Shou L. (2019). Heavy metal concentrations in commercial marine organisms from Xiangshan Bay, China, and the potential health risks. Mar. Pollut. Bull..

[63] Schümann K., Classen H.G., Dieter H.H., König J., Multhaup G., Rükgauer M., Summer K.H., Bernhardt J., Biesalski H.K. (2002). Hohenheim Consensus Workshop: Copper. Eur. J. Clin. Nutr..

[64] Velusamy A., Satheesh Kumar P., Ram A., Chinnadurai S. (2014). Bioaccumulation of heavy metals in commercially important marine fishes from Mumbai Harbor, India. Mar. Pollut. Bull..

[65] Ahmed M.K., Shaheen N., Islam M.S., Habibullah-al-Mamun M., Islam S., Mohiduzzaman M., Bhattacharjee L. (2015). Dietary intake of trace elements from highly consumed cultured fish (*Labeo rohita*, *Pangasius pangasius* and *Oreochromis mossambicus*) and human health risk implications in Bangladesh. Chemosphere.

[66] Barath Kumar S., Padhi R.K., Satpathy K.K. (2019). Trace metal distribution in crab organs and human health risk assessment on consumption of crabs collected from coastal water of South East coast of India. Mar. Pollut. Bull..

[67] Bebbington G.N., Mackay N.J., Chvojka R., Williams R.J., Dunn A., Auty E.H. (1977). Heavy metals, selenium and arsenic in nine species of Australian commercial fish. Mar. Freshw. Res..

[68] WHO (1989). Environmental Health Criteria. Heavy Metals—Environmental Aspects.

[69] Ersoy B., Çelik M. (2009). Essential elements and contaminants in tissues of commercial pelagic fish from the Eastern Mediterranean Sea. J. Sci. Food Agric..

[70] Kaimoussi A., Chafik A., Cheggour M., Mouzdahir A., Bakkas S. (2000). Variations saisonnières des concentrations en métaux (Cd, Cu, Zn, Fe et Mn) chez la moule *Mytilus galloprovincialis* du littoral de la région d’El Jadida (Maroc). Mar. Life.

[71] Bouthir F.Z., Chafik A., Benbrahim S., Souabi S., El Merdhy H., Messoudi A., Sifeddine M. (2004). Qualité physico-chimique des eaux côtières du littoral de la Wilaya du grand Casablanca (océan Atlantique marocain) utilisant la moule *Mytilus galloprovincialis* comme indicateur de la contamination métallique. Mar. Life.

[72] Aytekin T., Kargın D., Çoğun H.Y., Temiz Ö., Varkal H.S., Kargın F. (2019). Accumulation and health risk assessment of heavy metals in tissues of the shrimp and fish species from the Yumurtalik coast of Iskenderun Gulf, Turkey. Heliyon.

[73] Kargın F., Dönmez A., Çoğun H.Y. (2001). Distribution of heavy metals in different tissues of the shrimp *Penaeus semiculatus* and *Metapenaeus monocerus* from the Iskenderun Gulf, Turkey: Seasonal variations. Bull. Environ. Contam. Toxicol..

[74] Çoğun H., Yüzereroğlu T.A., Kargin F., Firat Ö. (2005). Seasonal variation and tissue distribution of heavy metals in shrimp and fish species from the Yumurtalik Coast of Iskenderun Gulf, Mediterranean. Bull. Environ. Contam. Toxicol..

[75] Canpolat Ö., Çalta M. (2003). Heavy metals in some tissues and organs of *Capoeta capoeta umbla* (Heckel, 1843) fish species in relation to body size, age, sex and seasons. Fresenius Environ. Bull..

[76] Zubcov E., Zubcov N., Ene A., Biletchi L. (2012). Assessment of copper and zinc levels in fish from freshwater ecosystems of Moldova. Environ. Sci. Pollut. Res..

[77] Ivanković D., Pavičić J., Erk M., Filipović-Marijić V., Raspor B. (2005). Evaluation of the Mytilus galloprovincialis Lam. digestive gland metallothionein as a biomarker in a long-term field study: Seasonal and spatial variability. Mar. Pollut. Bull..

[78] Ali A., Al-Abri E.S., Goddard J.S., Ahmed S.I. (2013). Seasonal variability in the chemical composition of ten commonly consumed fish species from Oman. J. Anim. Plant Sci..

[79] Arulkumar A., Paramasivam S., Rajaram R. (2017). Toxic heavy metals in commercially important food fishes collected from Palk Bay, Southeastern India. Mar. Pollut. Bull..

[80] Jarosz-Krzemińska E., Mikołajczyk N., Adamiec E. (2021). Content of toxic metals and As in marine and freshwater fish species available for sale in EU supermarkets and health risk associated with its consumption. J. Sci. Food Agric..

[81] Saeed S. (2013). Impact of environmental parameters on fish condition and quality in Lake Edku, Egypt. Egypt. J. Aquat. Biol. Fish..

[82] Ragi A.S., Leena P.P., Cheriyan E., Nair S.M. (2017). Heavy metal concentrations in some gastropods and bivalves collected from the fishing zone of South India. Mar. Pollut. Bull..

[83] Wang X.L., Wang Z.Q., Ma X.P., Subramanian S.V., Xie Z.J., Shang C.J., Li X.C. (2018). Analysis of impact toughness scatter in simulated coarse-grained HAZ of E550 grade offshore engineering steel from the aspect of crystallographic structure. Mater. Charact..

[84] Li J., Huang Z.Y., Hu Y., Yang H. (2013). Potential risk assessment of heavy metals by consuming shellfish collected from Xiamen, China. Environ. Sci. Pollut. Res..

[85] USEPA (2008). Integrated Risk Information System.

[86] Bratakos M.S., Lazos E.S., Bratakos S.M. (2002). Chromium content of selected Greek foods. Sci. Total Environ..

[87] Anandkumar A., Nagarajan R., Prabakaran K., Rajaram R. (2017). Trace metal dynamics and risk assessment in the commercially important marine shrimp species collected from the Miri coast, Sarawak, East Malaysia. Reg. Stud. Mar. Sci..

[88] Lingard S., Norseth T. (1979). Chromium. Hand Book on the Toxicology of Metals.

